# Supplementary material for: Identification of a Candidate Gene Set Signature for the Risk of Progression in IgM MGUS to Smoldering/Symptomatic Waldenström Macroglobulinemia (WM) by a Comparative Transcriptome Analysis of B Cells and Plasma Cells
Source: Cancers (Basel). 2021 Apr 12;13(8):1837. doi: 10.3390/cancers13081837 (PMC8070603; doi:10.3390/cancers13081837)
Supplement: Supplementary file 1 [file cancers-13-01837-s001.zip › cancers-1130262 Supplementary Material.doc]

Supplementary Material: Identification of a Candidate Gene Set Signature for the Risk of Progression in IgM MGUS to Smoldering/Symptomatic Waldenström Macroglobulinemia (WM) by a Comparative Transcriptome Analysis of B Cells and Plasma Cells

Alessandra Trojani, Barbara Di Camillo, Luca Emanuele Bossi, Livia Leuzzi, Antonino Greco,
Alessandra Tedeschi, Anna Maria Frustaci, Marina Deodato, Giulia Zamprogna, Alessandro Beghini and Roberto Cairoli

**Table S1.** DEGs in the comparison between WM vs. IgM MGUS vs. CTRLs in B cells grouped for different patterns.

| **Transmembrane Proteins** | | | | | | | | |
| --- | --- | --- | --- | --- | --- | --- | --- | --- |
| **Gene Symbol** | **Pattern** | **q Value** | **Mean WM** | **Mean MGUS** | **Mean CTRL** | **FC (*p* value)**  **WM vs. MGUS** | **FC (*p* value)**  **MGUS vs. CTRL** | **FC (*p* value)**  **WM vs. CTRL** |
| PTPRG | p1 | 0.007 | 4.19 | 2.8 | 2.32 | 2.61 (0.0000521) | 1.4 (0.0294557) | 3.66 (0.0000004) |
| MGAT3 | p1 | 0.01 | 6.56 | 5.89 | 5.8 | 1.59 (0.0002785) | 1.07 (0.618808) | 1.69 (0.0002612) |
| ATP1B1 | p1 | 0.014 | 8.34 | 7.06 | 6.59 | 2.43 (0.0047176) | 1.39 (0.3256879) | 3.37 (0.0001695) |
| GUCY2C | p1 | 0.017 | 7.24 | 6.15 | 6.02 | 2.12 (0.0014064) | 1.09 (0.7373912) | 2.32 (0.0017883) |
| PTPN4 | p1 | 0.018 | 4.85 | 4.18 | 4.08 | 1.6 (0.0007019) | 1.07 (0.595204) | 1.7 (0.0001499) |
| GPER | p1 | 0.021 | 6.96 | 5.48 | 5.34 | 2.81 (0.0000113) | 1.1 (0.5329286) | 3.09 (0.0002385) |
| CNR1 | p1 | 0.029 | 5.37 | 4.73 | 4.7 | 1.57 (0.0003542) | 1.02 (0.9141355) | 1.59 (0.0072828) |
| PTPN23 | p1 | 0.043 | 6.45 | 6.05 | 5.96 | 1.31 (0.007558) | 1.07 (0.6264284) | 1.4 (0.0124144) |
| **Hematopoietic Cell Lineage (hsa04640)** | | | | | | | | |
| **Gene symbol** | **Pattern** | **q Value** | **Mean WM** | **Mean MGUS** | **Mean CTRL** | **FC (*p* value)**  **WM vs. MGUS** | **FC (*p* value)**  **MGUS vs. CTRL** | **FC (*p* value)**  **WM vs. CTRL** |
| KITLG | p4 | 0.005 | 2.43 | 3.09 | 2.55 | −1.58 (0.0007285) | 1.45 (0.0105673) | −1.09 (0.1916959) |
| CD44 | p1 | 0.009 | 10.97 | 9.98 | 9.93 | 1.99 (0.0015137) | 1.04 (0.8718331) | 2.06 (0.0004416) |
| GP1BA  (CD42B) | p4 | 0.027 | 5.75 | 6.86 | 6.52 | −2.16 (0.0021354) | 1.26 (0.5621279) | −1.71 (0.139372) |
| IL2RA (CD25) | p1 | 0.03 | 6.88 | 5.72 | 5.69 | 2.23 (0.0044697) | 1.02 (0.9504548) | 2.27 (0.0011063) |
| ITGAM (CD11b) | p4 | 0.032 | 5.22 | 5.56 | 4.63 | −1.26 (0.0442824) | 1.9 (0.0000079) | 1.51 (0.0001444) |
| GP9/CD42a | p4 | 0.045 | 6.07 | 6.51 | 6.1 | −1.36 (0.0135909) | 1.33 (0.0525377) | −1.02 (0.8049944) |
| CD1D | p1 | 0.046 | 8.61 | 7.65 | 7.37 | 1.95 (0.011492) | 1.22 (0.3945029) | 2.37 (0.0000695) |
| CD4 | p4 | 0.048 | 3.62 | 3.88 | 3.42 | −1.2 (0.0446838) | 1.38 (0.0024367) | 1.15 (0.0161215) |
| **Cell adhesion Molecules, CAMs (hsa04514)** | | | | | | | | |
| **Gene Symbol** | **Pattern** | **q Value** | **Mean WM** | **Mean MGUS** | **Mean CTRL** | **FC (*p* value)**  **WM vs. MGUS** | **FC (*p* value)**  **MGUS vs. CTRL** | **FC (*p* value)**  **WM vs. CTRL** |
| IGHM | p1 | 0.001 | 13.71 | 12.82 | 12.78 | 1.84 (0.0000607) | 1.03 (0.8346248) | 1.9 (0.0000027) |
| CD86 | p4 | 0.009 | 5.53 | 7.08 | 6.54 | −2.93 (0.0000521) | 1.45 (0.2185342) | −2.02 (0.0171571) |
| CD226 | p4 | 0.014 | 3.64 | 4.89 | 4.63 | −2.37 (0.0015054) | 1.19 (0.6740898) | −1.99 (0.0669861) |
| HLA−A | p1 | 0.015 | 13.06 | 12.76 | 12.68 | 1.24 (0.0022758) | 1.05 (0.4643054) | 1.3 (0.0002518) |
| HLA−C | p1 | 0.016 | 12.12 | 11.78 | 11.69 | 1.27 (0.0130011) | 1.06 (0.4522633) | 1.35 (0.0028369) |
| CNTNAP2 | p2 | 0.018 | 3.68 | 4.41 | 4.88 | −1.66 (0.0121352) | −1.38 (0.1622985) | −2.29 (0.0003463) |
| GLG1 | p3 | 0.018 | 9.15 | 8.22 | 9.08 | 1.91 (0.0051446) | −1.81 (0.0116851) | 1.05 (0.5341155) |
| HLA−B | p1 | 0.019 | 13.15 | 12.91 | 12.87 | 1.18 (0.0065238) | 1.03 (0.641029) | 1.22 (0.0012654) |
| CEACAM1 (CD66a) | p3 | 0.019 | 5.57 | 5.13 | 5.29 | 1.36 (0.0006787) | −1.12 (0.1689084) | 1.21 (0.0056238) |
| CD34 | p2 | 0.019 | 5.36 | 5.8 | 6.27 | −1.36 (0.018989) | −1.38 (0.094386) | −1.88 (0.0023267) |
| HLA−G | p1 | 0.022 | 11.3 | 10.89 | 10.81 | 1.33 (0.0021553) | 1.05 (0.8626715) | 1.4 (0.0019539) |
| CDH2 | p2 | 0.027 | 2.99 | 3.36 | 3.42 | −1.29 (0.0100574) | −1.04 (0.7344842) | −1.34 (0.010577) |
| NRXN3 | p4 | 0.031 | 3.82 | 4.27 | 3.85 | −1.37 (0.0070539) | 1.34 (0.0206584) | −1.02 (0.7449887) |
| PTPRM | p4 | 0.035 | 3.43 | 3.9 | 3.36 | −1.39 (0.0111051) | 1.45 (0.0127689) | 1.05 (0.5732029) |
| ADAM23 | p2 | 0.036 | 4.14 | 4.84 | 6.13 | −1.62 (0.1457036) | −2.45 (0.0145243) | −3.95 (0.0000281) |
| HLA−E | p1 | 0.038 | 12.04 | 11.69 | 11.53 | 1.27 (0.0171861) | 1.12 (0.295144) | 1.42 (0.0007756) |
| CD6 | p4 | 0.042 | 4.63 | 5.25 | 4.77 | −1.54 (0.0090559) | 1.39 (0.0798046) | −1.1 (0.4155172) |
| CD58 | p3 | 0.045 | 5.9 | 5.17 | 5.28 | 1.65 (0.0009001) | −1.08 (0.5336323) | 1.54 (0.0012058) |
| NEGR1 | p4 | 0.047 | 2.22 | 2.59 | 2.58 | −1.3 (0.0175088) | 1.01 (0.9313534) | −1.28 (0.0109034) |
| CDHR3 | p2 | 0.048 | 4.82 | 5.31 | 6.29 | −1.4 (0.1418814) | −1.98 (0.0280131) | −2.77 (0.0014673) |
| **Focal Adhesion (hsa 04510)** | | | | | | | | |
| **Gene Symbol** | **Pattern** | **q Value** | **Mean WM** | **Mean MGUS** | **Mean CTRL** | **FC (*p* value)**  **WM vs. MGUS** | **FC (*p* value)**  **MGUS vs. CTRL** | **FC (*p* value)**  **WM vs. CTRL** |
| RAPGEF1 | p3 | 0.02 | 7.39 | 6.41 | 6.71 | 1.98 (0.0003335) | −1.24 (0.225606) | 1.6 (0.0040966) |
| ITGB5 | p4 | 0.023 | 5.62 | 6.55 | 6.04 | −1.9 (0.0025264) | 1.42 (0.2055098) | −1.34 (0.185358) |
| ITGA2B | p4 | 0.024 | 6.44 | 7.81 | 7.51 | −2.58 (0.0020097) | 1.23 (0.6479419) | −2.09 (0.0797696) |
| ITGB3 | p4 | 0.024 | 5.24 | 7 | 7.22 | −3.39 (0.0030369) | −1.16 (0.8174328) | −3.94 (0.0320509) |
| IGF1R | p2 | 0.027 | 4.29 | 4.93 | 5.52 | −1.56 (0.0126827) | −1.5 (0.1124533) | −2.33 (0.0046749) |
| THBS1 | p4 | 0.028 | 5.26 | 6.74 | 6.39 | −2.78 (0.0023687) | 1.27 (0.5674047) | −2.2 (0.0327858) |
| PDGFA | p4 | 0.03 | 4.71 | 5.44 | 5.2 | −1.66 (0.0083051) | 1.18 (0.4200568) | −1.4 (0.0215361) |
| CAV1 | p2 | 0.035 | 4.47 | 5.63 | 5.9 | −2.24 (0.0076386) | −1.2 (0.5759653) | −2.68 (0.0019275) |
| ACTN1 | p2 | 0.043 | 6.15 | 7.39 | 7.81 | −2.36 (0.0126826) | −1.34 (0.4474237) | −3.15 (0.0023684) |
| VAV3 | p2 | 0.05 | 6.71 | 7.2 | 7.7 | −1.4 (0.0423252) | −1.41 (0.034547) | −1.98 (0.0000693) |
| PTK2 | p2 | 0.05 | 2.79 | 3.47 | 3.61 | −1.6 (0.0233812) | −1.1 (0.7004071) | −1.76 (0.0108199) |
| **Adherens Junctions (hsa04520)** | | | | | | | | |
| **Gene Symbol** | **Pattern** | **q Value** | **Mean WM** | **Mean MGUS** | **Mean CTRL** | **FC (*p* value)**  **WM vs. MGUS** | **FC (*p* value)**  **MGUS vs. CTRL** | **FC (*p* value)**  **WM vs. CTRL** |
| PTPRJ | p1 | 0.007 | 9.88 | 8.86 | 8.69 | 2.03 (0.0011401) | 1.12 (0.5509737) | 2.27 (0.0000016) |
| TJP1 | p4 | 0.015 | 2.82 | 3.23 | 3.11 | −1.33 (0.002953) | 1.09 (0.4634889) | −1.22 (0.0371856) |
| SSX2IP | p2 | 0.017 | 4.86 | 5.67 | 6.23 | −1.75 (0.0169615) | −1.47 (0.1119827) | −2.58 (0.0000218) |
| PTPN1 | p1 | 0.047 | 9.02 | 8.45 | 8.43 | 1.48 (0.0092316) | 1.02 (0.8949732) | 1.51 (0.0012067) |
| **Wnt/βcatenin Signaling Pathway (hsa04310)** | | | | | | | | |
| **Gene Symbol** | **Pattern** | **q Value** | **Mean WM** | **Mean MGUS** | **Mean CTRL** | **FC (*p* value)**  **WM vs. MGUS** | **FC (*p* value)**  **MGUS vs. CTRL** | **FC (*p* value)**  **WM vs. CTRL** |
| PRKCA | p4 | 0.004 | 3.98 | 4.91 | 4.33 | −1.91 (0.0000518) | 1.49 (0.0058912) | −1.28 (0.0044) |
| MARCKS | p3 | 0.017 | 9.41 | 8.59 | 8.64 | 1.76 (0.0012965) | −1.03 (0.8409818) | 1.71 (0.0000996) |
| CAMK2G | p3 | 0.026 | 6.99 | 6.5 | 7.11 | 1.4 (0.0017322) | −1.52 (0.0006227) | −1.08 (0.2743517) |
| RYK | p3 | 0.032 | 7.65 | 7.25 | 7.35 | 1.32 (0.000791) | −1.07 (0.5246633) | 1.23 (0.0404351) |
| PSEN1 | p3 | 0.038 | 6.33 | 6.01 | 6.61 | 1.25 (0.0324061) | −1.51 (0.0016432) | −1.21 (0.0215568) |
| LEF1 | p2 | 0.041 | 5.24 | 5.82 | 7.19 | −1.5 (0.1895199) | −2.58 (0.0095905) | −3.86 (0.0001365) |
| WNT3 | p1 | 0.041 | 5.16 | 4.03 | 3.93 | 2.19 (0.001227) | 1.07 (0.7555602) | 2.35 (0.0013753) |
| PPP3CA | p3 | 0.05 | 9.6 | 9.02 | 9.45 | 1.49 (0.0025554) | −1.35 (0.0318975) | 1.11 (0.3029499) |
| **Calcium Signaling Pathway (hsa04020)** | | | | | | | | |
| **Gene Symbol** | **Pattern** | **q Value** | **Mean WM** | **Mean MGUS** | **Mean CTRL** | **FC (*p* value)**  **WM vs. MGUS** | **FC (*p* value)**  **MGUS vs. CTRL** | **FC (*p* value)**  **WM vs. CTRL** |
| ITPR3 | p3 | 0.006 | 7.9 | 6.98 | 7.27 | 1.89 (0.0000088) | −1.22 (0.13157) | 1.55 (0.0019284) |
| ITPR1 | p2 | 0.014 | 7.86 | 8.49 | 8.89 | −1.55 (0.0004027) | −1.32 (0.0639881) | −2.05 (0.000294) |
| SLC8A1 | p4 | 0.015 | 4.23 | 5.81 | 5.35 | −2.99 (0.000375) | 1.38 (0.2175169) | −2.17 (0.0004085) |
| ATP2A2 | p3 | 0.017 | 7.89 | 6.88 | 7.75 | 2.01 (0.0008872) | −1.82 (0.0047601) | 1.1 (0.3886408) |
| TRPC1 | p2 | 0.017 | 3.58 | 4.15 | 4.41 | −1.48 (0.003731) | −1.2 (0.2264183) | −1.77 (0.0003891) |
| ORAI2 | p3 | 0.025 | 8.43 | 7.79 | 7.91 | 1.56 (0.0017961) | −1.09 (0.6776486) | 1.43 (0.0645664) |
| MYLK | p2 | 0.026 | 6.21 | 7.31 | 7.88 | −2.14 (0.0207183) | −1.48 (0.2861703) | −3.17 (0.0003539) |
| CACNA1D | p1 | 0.029 | 4.83 | 3.99 | 3.43 | 1.79 (0.0037504) | 1.47 (0.0581134) | 2.64 (0.0001954) |
| TPCN1 | p3 | 0.045 | 7.04 | 6.49 | 6.77 | 1.47 (0.0057479) | −1.21 (0.1952122) | 1.21 (0.0793209) |
| SLC8A3 | p4 | 0.047 | 5.1 | 5.38 | 4.82 | −1.22 (0.049277) | 1.48 (0.0041472) | 1.21 (0.0438423) |
| ADRB2 | p1 | 0.047 | 7.33 | 6.98 | 6.13 | 1.28 (0.2257776) | 1.8 (0.0048193) | 2.31 (0.0000031) |
| **BCR Signaling Pathway (hsa04662)** | | | | | | | | |
| **Gene Symbol** | **Pattern** | **q Value** | **Mean WM** | **Mean MGUS** | **Mean CTRL** | **FC (*p* value)**  **WM vs. MGUS** | **FC (*p* value)**  **MGUS vs. CTRL** | **FC (*p* value)**  **WM vs. CTRL** |
| MAP4K1 | p1 | 0.03 | 8.76 | 8.36 | 8.19 | 1.32 (0.0167919) | 1.12 (0.4753177) | 1.48 (0.0005974) |
| CD79A | p3 | 0.041 | 10.65 | 10.05 | 10.2 | 1.52 (0.0006752) | −1.11 (0.3832879) | 1.36 (0.0129936) |
| CBLB | p3 | 0.043 | 7.55 | 6.59 | 6.84 | 1.94 (0.0006278) | −1.19 (0.3297732) | 1.63 (0.0092351) |
| SYK | p3 | 0.044 | 8.47 | 7.54 | 8.16 | 1.91 (0.0126325) | −1.54 (0.1214568) | 1.24 (0.175708) |
| RASGRP3 | p3 | 0.045 | 4.57 | 4.47 | 5.6 | 1.07 (0.6872506) | −2.19 (0.0014631) | −2.05 (0.0021184) |
| PIK3AP1 | p2 | 0.047 | 10.15 | 10.24 | 10.84 | −1.06 (0.5648952) | −1.51 (0.0016433) | −1.61 (0.0002061) |
| SOS1 | p3 | 0.048 | 6.93 | 6.16 | 6.4 | 1.7 (0.0040252) | −1.18 (0.3484591) | 1.44 (0.0107719) |

| **Complement and Coagulation Cascade (hsa04610) and Platelet Activation (hsa 04611)** | | | | | | | | |
| --- | --- | --- | --- | --- | --- | --- | --- | --- |
| **Gene Symbol** | **Pattern** | **q Value** | **Mean WM** | **Mean MGUS** | **Mean CTRL** | **FC (*p* value)**  **WM vs. MGUS** | **FC (*p* value)**  **MGUS vs. CTRL** | **FC (*p* value)**  **WM vs. CTRL** |
| P2RY12 | p4 | 0.007 | 2.49 | 4.36 | 4.1 | −3.64 (0.0019318) | 1.2 (0.7380937) | −3.04 (0.0176512) |
| VWF | p4 | 0.015 | 4.73 | 5.47 | 5.31 | −1.67 (0.000491) | 1.12 (0.6522255) | −1.49 (0.0989058) |
| TFPI | p4 | 0.019 | 1.99 | 2.59 | 2.55 | −1.52 (0.0025791) | 1.03 (0.9188159) | −1.48 (0.1281706) |
| PTGIR | p4 | 0.023 | 5.82 | 6.38 | 5.84 | −1.47 (0.0008177) | 1.45 (0.0109416) | −1.01 (0.9173999) |
| RAP1A | p4 | 0.032 | 4.08 | 4.36 | 3.72 | −1.21 (0.028803) | 1.57 (0.0002216) | 1.29 (0.0058867) |
| SERPINE1 | p4 | 0.043 | 4.54 | 5.05 | 4.79 | −1.43 (0.001514) | 1.2 (0.2924057) | −1.19 (0.2546245) |
| **Cytoline−Cytokine Receptor Interacions (hsa04060) and IL−17 Signaling Pathway (hsa04657)** | | | | | | | | |
| **Gene Symbol** | **Pattern** | **q Value** | **Mean WM** | **Mean MGUS** | **Mean CTRL** | **FC (*p* value)**  **WM vs. MGUS** | **FC (*p* value)**  **MGUS vs. CTRL** | **FC (*p* value)**  **WM vs. CTRL** |
| PF4 | p4 | 0.005 | 7.86 | 10.3 | 9.88 | −5.41 (0.0000042) | 1.33 (0.5988193) | −4.05 (0.0194731) |
| PPBP | p4 | 0.011 | 8.98 | 11.67 | 11.51 | −6.48 (0.0000047) | 1.12 (0.8174824) | −5.8 (0.0038281) |
| TNFRSF10A | p3 | 0.013 | 6.85 | 6.06 | 6.49 | 1.73 (0.0003323) | −1.35 (0.047072) | 1.28 (0.0200506) |
| PF4V1 | p4 | 0.017 | 3.38 | 5.42 | 4.83 | −4.11 (0.0005975) | 1.5 (0.4674524) | −2.73 (0.0535646) |
| IL17RB | p1 | 0.018 | 7.62 | 5.51 | 5.3 | 4.34 (0.0001234) | 1.16 (0.7681523) | 5.02 (0.0003613) |
| BMPR2 | p1 | 0.023 | 7.56 | 6.5 | 6.49 | 2.09 (0.0039867) | 1.01 (0.9711482) | 2.1 (0.0001393) |
| **JAK−STAT Signaling Pathway (hsa04630)** | | | | | | | | |
| **Gene Symbol** | **Pattern** | **q Value** | **Mean WM** | **Mean MGUS** | **Mean CTRL** | **FC (*p* value)**  **WM vs. MGUS** | **FC (*p* value)**  **MGUS vs. CTRL** | **FC (*p* value)**  **WM vs. CTRL** |
| IL21R | p2 | 0.014 | 5.83 | 6.8 | 7.27 | −1.97 (0.0015957) | −1.39 (0.1125611) | −2.72 (0.0000364) |
| IL4R | p2 | 0.017 | 6.02 | 6.84 | 7.66 | −1.78 (0.0280912) | −1.76 (0.0738095) | −3.13 (0.0002308) |
| IL13RA1 | p2 | 0.026 | 4.79 | 5.75 | 6.05 | −1.95 (0.0018858) | −1.23 (0.4108191) | −2.39 (0.0019291) |
| STAT3 | p3 | 0.029 | 7.96 | 7.36 | 7.52 | 1.52 (0.0007817) | −1.11 (0.3467457) | 1.36 (0.0040669) |
| BCL2 | p3 | 0.032 | 9.76 | 8.74 | 8.84 | 2.02 (0.0025084) | −1.07 (0.7719439) | 1.89 (0.0044267) |
| SOS1 | p3 | 0.048 | 6.93 | 6.16 | 6.4 | 1.7 (0.0040252) | −1.18 (0.3484591) | 1.44 (0.0107719) |
| **FOXO Signaling Pathway (hsa04068) and TGF−β Signaling Pathway (hsa04350)** | | | | | | | | |
| **Gene Symbol** | **Pattern** | **q Value** | **Mean WM** | **Mean MGUS** | **Mean CTRL** | **FC (*p* value)**  **WM vs. MGUS** | **FC (*p* value)**  **MGUS vs. CTRL** | **FC (*p* value)**  **WM vs. CTRL** |
| CDKN1B | p1 | 0.01 | 10.14 | 9.55 | 9.55 | 1.5 (0.0002543) | 1 (0.980112) | 1.5 (0.00002) |
| SMAD6 | p4 | 0.012 | 3.69 | 4.1 | 3.46 | −1.33 (0.0032135) | 1.55 (0.0002647) | 1.17 (0.0261903) |
| IL6 | p1 | 0.017 | 8.86 | 7.45 | 7.32 | 2.65 (0.0037778) | 1.1 (0.8348148) | 2.91 (0.012244) |
| RAG1 | p2 | 0.025 | 4.06 | 5.03 | 6.04 | −1.96 (0.0126499) | −2.01 (0.0079946) | −3.94 (0.0000128) |
| EP300 | p3 | 0.031 | 8.6 | 7.94 | 8.51 | 1.58 (0.0049924) | −1.48 (0.0291049) | 1.06 (0.5658925) |
| SMAD3 | p2 | 0.031 | 5.2 | 5.68 | 6.07 | −1.4 (0.0174981) | −1.31 (0.126409) | −1.84 (0.0015276) |
| FOXO3 | p1 | 0.043 | 6.78 | 6.2 | 6.14 | 1.49 (0.0097111) | 1.04 (0.8000652) | 1.55 (0.0023117) |
| TGFB2 | p2 | 0.05 | 2.89 | 3.23 | 3.23 | −1.26 (0.014168) | −1 (0.9833347) | −1.27 (0.0070939) |
| **NFKB Signaling Pathway (hsa04064)** | | | | | | | | |
| **Gene Symbol** | **Pattern** | **q Value** | **Mean WM** | **Mean MGUS** | **Mean CTRL** | **FC (*p* value)**  **WM vs. MGUS** | **FC (*p* value)**  **MGUS vs. CTRL** | **FC (*p* value)**  **WM vs. CTRL** |
| PRKCQ | p2 | 0.017 | 4.59 | 5.28 | 5.7 | −1.62 (0.0063396) | −1.34 (0.1405202) | −2.16 (0.0002679) |
| NFKBIZ | p3 | 0.018 | 10.82 | 9.88 | 9.88 | 1.91 (0.003466) | −1 (0.9994712) | 1.91 (0.0007203) |
| XIAP | p3 | 0.02 | 8.14 | 7.21 | 7.71 | 1.91 (0.002152) | −1.42 (0.0756072) | 1.34 (0.0091811) |
| RIPK1 | p3 | 0.031 | 6.17 | 5.55 | 6.03 | 1.54 (0.0082501) | −1.39 (0.0520738) | 1.1 (0.2335854) |
| CFLAR | p3 | 0.031 | 8.16 | 7.48 | 7.65 | 1.6 (0.0017398) | −1.12 (0.4044885) | 1.43 (0.0026323) |
| CRKL | p3 | 0.032 | 8.63 | 8.11 | 8.63 | 1.43 (0.0050829) | −1.43 (0.0133642) | −1 (0.9809056) |
| **p53 Signaling Pathway (hsa04115)** | | | | | | | | |
| **Gene Symbol** | **Pattern** | **q Value** | **Mean WM** | **Mean MGUS** | **Mean CTRL** | **FC (*p* value)**  **WM vs. MGUS** | **FC (*p* value) MGUS vs. CTRL** | **FC (*p* value)**  **WM vs. CTRL** |
| CD82 | p1 | 0.017 | 8.64 | 8.06 | 7.75 | 1.49 (0.0049218) | 1.24 (0.1348033) | 1.85 (0.0000489) |
| TP73 | p1 | 0.018 | 4.4 | 4.07 | 3.83 | 1.25 (0.009593) | 1.19 (0.0485147) | 1.49 (0.0000216) |
| CHEK1 | p2 | 0.042 | 4.81 | 5.26 | 6.32 | −1.36 (0.2432231) | −2.09 (0.0159117) | −2.85 (0.0001151) |

Gene symbol is reported together with the pattern of expression (p1–p4), the q-value of the SAM statistics across the three groups, the average expression in WM, IgM MGUS and CTRL groups and the pair−wise Fold Changes (*p*-value of the SAM statistics between the two groups in parenthesis).

**Table S2** (separate excel file “Table S2 PatternsCD19”): Main patterns of differential expression for CD19 cells.

**Table S3** (separate excel file “Table S3 PatternsCD1138”): Main patterns of differential expression for CD138 cells.
